# An extended Theory of Planned Behavior in explaining intention toward sustainable forest management: Evidence from COVID 19 Pandemic from Bali, Indonesia

**DOI:** 10.12688/f1000research.158455.4

**Published:** 2026-04-06

**Authors:** Shine Pintor Siolemba Patiro, Kresno Agus Hendarto, Dian Charity Hidayat, Lukas Rumboko Wibowo, Digby Race, I Wayan Widhana Susila, Sutrihadi Sutrihadi, Krisdianto Sugiyanto, Gerson Ndawa Njurumana, Hani Sitti Nuroniah, Dewi Ratna Kurniasari, V. Rachmadi Parmono, Atfi Indriany Putri, Abdurakhman Abdurakhman, Tri Astuti Wisudayati, Ramawati Ramawati, Yudha Satria Aji Pratama

**Affiliations:** 1 Indonesia Open University, Jakarta, Indonesia; 2 National Research and Innovation Agency, Indonesia, Indonesia; 3 The University of the South Pacific, Suva, Fiji; 4 Ministry of Environment and Forestry, Jakarta, Indonesia; 5 Atma Jaya Catholic University, Jakarta, Indonesia; 6 Gadjah Mada University, Sleman, Indonesia; 7 Sepuluh Nopember Institute of Technology, Surabaya, Indonesia

**Keywords:** Theory of planned behavior”, COVID-19, “return migrants”, “sustainable forest management”, “Tri Hita Karana”

## Abstract

**Background:**

The COVID-19 pandemic has generated significant impacts on the forestry sector. Employment layoffs have led to an increase in return migration, resulting in additional labor supply and heightened family economic burdens. This research employs the Theory of Planned Behaviour (TPB) framework to examine and predict sustainable forest management practices among families managing customary forests and village forests in Bali.

**Methods:**

Purposive sampling was used to collect data from 71 managers of customary forests and village forests in Tenganan and Wanagiri. Partial least square-structural equation modelling (PLS-SEM) was used to analyze the acquired data.

**Results:**

The findings demonstrated that TPB can explain the sustainable forest management. The incorporation of an additional construct, *Tri Hita Karana* (THK), enhanced the model’s predictive power for both managerial intentions and behaviors in sustainable forest management. Specifically, *THK* influences management intentions through the mediation of attitudes, subjective norms, and perceived behavioral control.

**Conclusions:**

This study established that *THK*, a fundamental value system in Balinese society, serves as an antecedent predictor of behavioral intentions toward sustainable forest management. The relationship between *THK* and sustainable forest management intentions is mediated by attitudes, subjective norms, and perceived behavioral control. This research makes significant theoretical and managerial contributions. First, it validates the established TPB framework within the context of COVID-19’s impact in Bali. Additionally, it provides scholars with insights for identifying other potential constructs that may influence forest land managers’ behavior.

## Introduction

At the end of 2019, the World Health Organization (WHO) for the first time recognized a COVID-19 case in China and deemed it as a global pandemic on March 11, 2020. “Over the past two weeks, the number of cases outside China has increased 13-fold, and the number of affected countries has increased 3-fold,” said WHO Secretary-General Tedros Adhanom Ghebreyesus ( Dzulfaroh 2021).

To limit the spread of COVID-19, all governments globally took drastic measures by locking down entire countries or most affected cities and towns and banning outsiders from entering their countries ( Fotiadis et al. 2021). As a result, patterns of social, economic, and human behavior alter quickly and dramatically ( Cooke et al. 2021). This behavior change has resulted in reduced greenhouse gas emissions, air pollution, noise pollution, and waste which makes beaches in different countries cleaner ( Ming et al. 2020; Bao and Zhang 2020; Dantas et al. 2020; Maji et al. 2021; El-Sayed et al. 2021; Rahman et al. 2021). However, it has also resulted in weakly enforced regulations and environmental law handling ( Corlett et al. 2020). Comparing the same period in 2019 to all tropical areas, deforestation increased by 63% to 136% ( Brancalion et al. 2020). In Gundaki Province, Nepal, COVID-19 has suspended all types of forestry and ecotourism-based businesses, research, and monitoring activities. It has also led to a drastic increase in illegal logging and poaching both inside and outside protected areas, a significant reduction in the income of the middle and lower classes, and an increase in rural and urban poverty ( Laudari et al. 2021). Besides that, extensive protective measures such as mask and glove use have increased organic and inorganic waste in the environment ( Zambrano-Monserrate et al. 2020).

In Indonesia, President Joko Widodo and Minister of Health Terawan announced the Covid 19 case at a press conference at the Presidential Palace on March 2, 2020. Two patients with Covid 19 were confirmed. Patient 1 is a 31-year-old woman while Patient 2 was a 64-year-old woman. They are a mother and daughter who live in one house in Depok, West Java. Even though at the beginning of the pandemic the Government received much criticism from the public, on May 25, 2022, at the 7th Global Platform for Disaster Risk Reduction 2022 in Bali, Indonesia was highly appreciated by the President of the UN General Assembly, Abdulla Shahid, in his remarks at the event ( BNPB 2022).

Tourism is the backbone of Bali’s economy. When the COVID-19 pandemic struck, its impact was significant. In the Bali Economic and Investment Forum on April 8, 2021, the Head of the Bali Tourism Office stated, “Three thousand employees were laid off and consequently increased the unemployment in Bali. While usually, it has the lowest rate nationally, it is now in 18th position” ( CNN 2021). The open unemployment rate in February 2020 was 1.3%, which increased to 5.4% in February 2021 and fell slightly to 5.3% in February 2022. The poverty rate increased, in March 2020 it was 165.19 (3.8%) while in March 2021, it was 201.97 (4.5%) ( BPS 2022). Like in other countries the laid-off employees generally returned to their hometowns and were involved in work.

Previous researchers (i.e., Pramana et al. 2022; Khalid et al. 2021; Zenker and Kock 2020; Higgins-Desbiolles 2020; Guridno and Guridno 2020; Baum and Hai 2020; Qiu et al. 2020; Nicola et al. 2020; Foo et al. 2021; Qiu et al. 2021; Škare et al. 2021; Abbas et al. 2021; Zhang et al. 2021; Fotiadis et al. 2021; Bae and Chang 2021) have studied the COVID-19 pandemic and the impact on tourism. Using web-based bibliometric analysis (biblioshiny) on the impact of the COVID-19 pandemic on the forestry sector, Jayasundara et al. (2024) identified that “deforestation” appears most frequently. Subsequently, “conservation”, “management”, “urban”, “recreation”, “wildlife”, “market”, “product”, “climate change”, “ecosystem services” come next. Interestingly, there has been an increase in forest recreation (especially urban forests) in such developed countries as America ( Grima et al. 2020; Ferguson et al. 2022), Japan ( Yamazaki et al. 2021), Germany ( da Schio et al. 2021). Urban forests have been favored because of they are close to their homes. In addition to urban forests, undeveloped forests (wilderness areas) are other favorite destinations since the exercise of social distancing regulations. Therefore, they visit isolated tourist locations with only few visitors. On the other hand, the number of retiree visitors (who are older), international visitors, and those from distant locations has decreased. Likewise, the intensity of activities forest conservation and management has decreased because in general the government has allocated the budget mostly to deal with COVID-19. In other words, the budget for conservation and forest management activities has been cut significantly. Consequently, such illegal activities as deforestation, land encroachment, and poaching have increased ( Brancalion et al. 2020). With the transit point in Bangladesh, Rahman et al. (2021) revealed that, hunting and trade of wild animals has increased in India, Nepal, Myanmar, Thailand and China. The people whose lives depend on forests face serious challenges. The restrictions imposed has resulted in income decline. For instance, in Indonesia, limited accessibility has resulted in the decline in honey harvest and income ( Njurumana et al. 2021). The decline in demand for agroforestry products has consequently resulted in reduced income ( Pieter et al. 2022). During COVID-19, bamboo craftsmen in Gunung Kidul Yogyakarta have earned up to 23.5% lower income. Consequently, they do such other jobs as farming, hunting, and selling food ( Utomo et al. 2022). Likewise, a systematic evaluation using 62 indicators has revealed that COVID 19 has caused a significant decrease in production marketing, income, and social interaction for forest farmers in Sikka, East Nusa Tenggara ( Njurumana et al. 2025). The decline of ecotourism due to the decreased number of distant and international visitors has decreased the income of the communities around ecotourism locations ( Kalema-zikusoka 2021; Rahaman et al. 2021). Furthermore, research on forest cover showed that during the pandemic tropical forest cover on small inhabited islands has declined. For example, forest cover on Mansinam Island decreased by 4.3%, wasteland increased by 80.6%, agricultural land increased by 75.3%, and shrubs increased by 54.9%. Another finding is that 78.9% of total deforestation has resulted from forest conversion to wasteland and agricultural land ( Hematang et al., 2025).

Reviewing these literatures, we found that significant gap, especially on migrant-receiving family (1) most of the studies were based on assumptions (valid for scenario analysis) and generally, they used secondary data or online survey, instead of the actual data taken in the field; (2) most of these studies did not focus on migrants affected by layoffs and acceptance of the hometown of the returning migrants; and (3) families of migrant workers who have been laid off were affected by both internal factor (biological, psychological, and social) and external factor (assistance from the central and local governments).

This study aims to fill that gap. **First**, this study focuses on the hometown of the migrants by focusing on the behavior of migrant-receiving families (especially managers of customary forests land and village forests). **Second**, this study gathers field data. This study collects information directly from the source to ensure more authentic and representative insights, which can objectively reveal real-world phenomena and capture the possibly overlooked complexities that are relevant to the study questions or hypotheses. **Third**, this study examines internal factors using Theory of planned behavior (TPB) to understand behavior of migrant receiving families. Gao et al. (2017) stated that although TPB has been widely used in predicting individual pro-environmental behavior, there are 2 limitations. The limitation is that TPB is a theory of self-interest and all variables in TPB are rational predictors ( Bertoldo and Castro 2016). In other words, TPB assumes that human behavior is simple, so that people make decisions using rational thinking; in fact human behavior is very complex ( Ajzen 1991; Petty and Cacioppo 1986). Thus, to enhance the ability of TPB in explaining and predicting intentions and pro-environmental behavior, it is necessary to consider additional variables for inclusion in the model ( Conner and Armitage 1998). This study has expanded the TPB framework by including *THK* (as a value) construct to measure the impact on the behavior of customary forest and village forest managers.

TPB is the most frequently cited theory to explain human behavior ( Sussman and Gifford 2019). Numerous empirical studies have examined and validated this theory, which has been found to be an effective explanation for a range of pro-environmental behaviors ( Sarkis 2017; Du and Pan 2021). This theory is a development of the theory of reasoned action and was first proposed by Icek Ajzen in 1985. This theory states that human behavior is guided by 3 kinds of considerations, namely behavioral belief, normative belief, and control belief, which in turn produce specific outcomes such as attitudes toward behavior, subjective norm (SN), and perceived behavioral control (PBC) ( Yadav and Pathak 2017).

Values influence behavior when they are relevant to the context and important to the individual ( Schwartz 1992). Individuals hold a relatively stable set of values that are internalized from early life stages and change little thereafter ( Schwartz 1994). In other words, values are used to characterize cultural groups, societies, and individuals, to explain the motivational basis of attitudes and behavior ( Schwartz 2012). Schwartz’s approach is crucial for social-psychological studies for various reasons. First, it directly deals with theory, and its fundamental components are incorporated into early social scientific research ( Desender et al. 2011; Ahmad et al. 2020). Second, the framework utilizes value dimensions measurements that are consistent across cultures ( Burgess and Steenkamp 2006; Schwartz 2006; Ahmad et al. 2020).

Stern et al. (1993) proposed three value orientations that were pertinent to consumers’ environmental concerns as an early application of Schwartz’s value theory: self-interest, altruism toward other humans, and altruism toward other species and the biosphere. Later, Stern and Dietz (1994) asserted that a person’s perspective about themselves (egoistic value orientation), other people (altruistic value orientation), or plants and animals (biospheric value orientation) will determine how important they view environmental issues.

The term *THK* derives from the words “Tri” which means three, “Hita” which means happiness. and “Karana” which means cause. Therefore, lexically the term means three causes of happiness creation ( Yhani and Supastri 2020). Some examples of the implementing of our gratitude to God include (1) with *sradha* (belief or trust) and *bhakti* (activity of getting closer to God) giving *yadny*a (divine service) and praying to God. Doing *Punia* (offerings) without any strings attached, doing *tirtta yatra* (holy journey) to places that can lead to their sacred values; (2) Caring for others, especially to a relative (fellow) hit by a disaster. As role model that illuminates others, at least we must be a torch for ourselves first by diligently talking about virtue while taking real action; (3) The natural surroundings or our environment is our closest mirror of caring for nature. The environment looks beautiful, clean, and neatly arranged, which means that we can realize one of the *THK.* In the Bhagawadgita it is said that “ *Satatam kirtayatom mam. Yatantas ca drsha vrtatah. Namasyantas ca mam bhatya. Ni tyayuktah upsate*” (IX.14) (Always exclusively praise Me and do the duty of service uninterruptedly. You who worship me unceasingly and with eternal devotion are close to Me) ( Budiastika 2022).

Thus, the aim of this study is to improve understanding of the behavior of the migrant-receiving families. The word migrants in this study exclusively refers to return-migrant, namely those who inevitable return to their hometowns as a result of layoffs due to COVID-19. In other words, this study aims to investigate of the insignificant impact of COVID-19 pandemic-related damage on the management of customary forests and village forests in Bali. This study proposes two questions. They are (1) what internal factors significantly influence the behavior of customary/village forest managers; and (2) how these factors shape the behavior of the managers of customary forests and/or village forests.

This article illustrates the process as follows. The second section describes context, sample, measurement, and analysis of data. We present results and discussion in the third section. Finally, the fourth section presents conclusions, limitations, and suggestions.

## Methods

### Context

**Figure 1.**
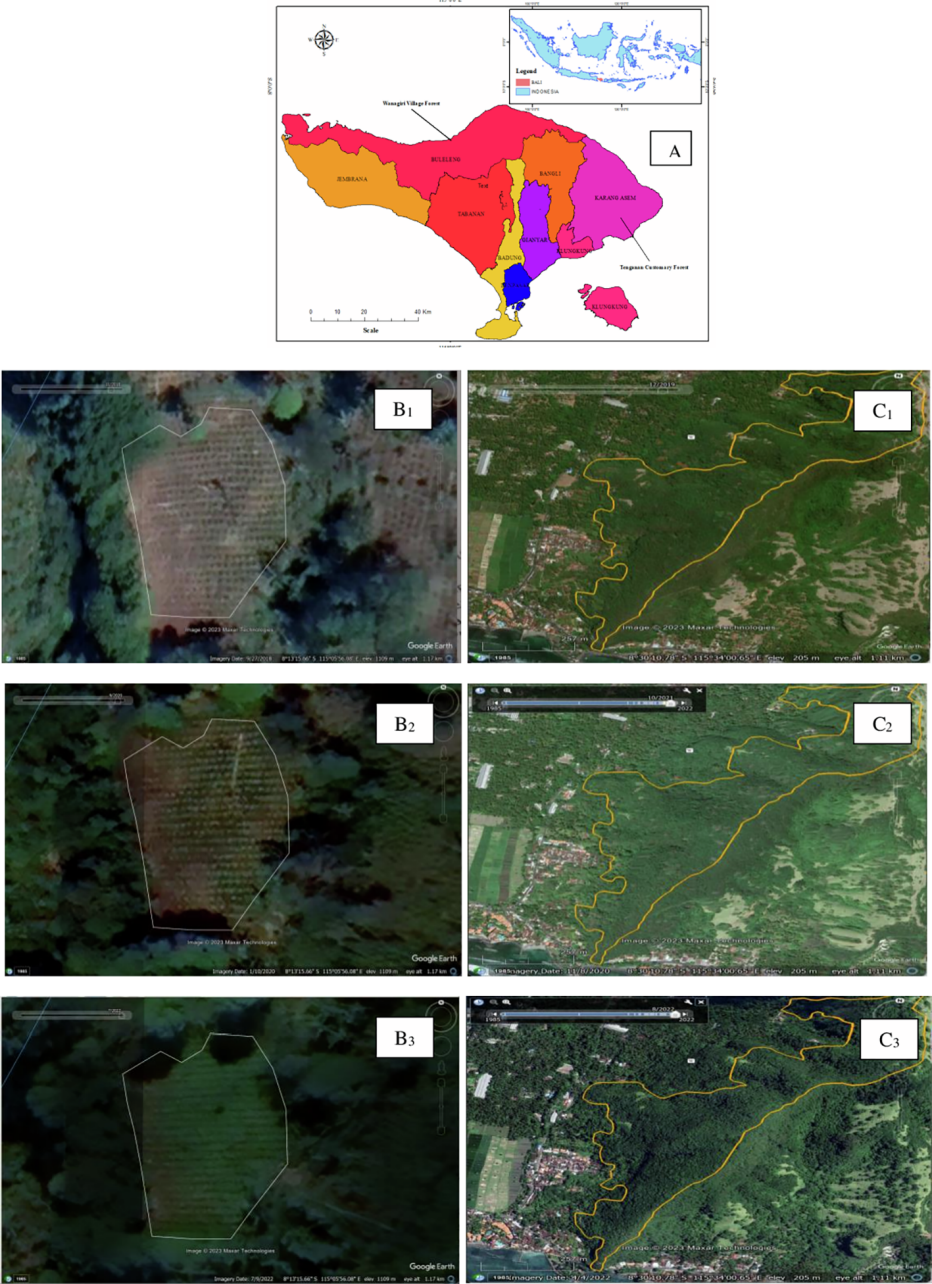
Study area, Village forest and Customary Forest land cover. Note: Bali Island (A) (Source: BPS, 2023); Village forest land cover in Wanagiri 2018 (B1), 2020 (B2), and 2022 (B3); Customary forest land cover in Tenganan 2018 (C1), 2020 (C2), and 2022 (C3).

The study was conducted in two villages, namely Tenganan Village and Wanagiri Village. The two villages were chosen because: 1) Both villages represent two dominant management models. The forest land in Tenganan is communally owned, which is a model of prototypically traditional customary management, while the forest land in Wanagiri Village is state-owned, which is a model of state-supported community forest managemen; (2) Managers of customary forests and village forests have been incorporated into forest farmer group established before the COVID-19 pandemic. Simply said, forest farmer groups in Tenganan and Wanagiri have been well-established before the COVID-19 pandemic. Therefore, we could observe the resilience of both institutional structures and the behavior of migrant recipient families (especially those who manage customary forest and village forest land); (3) there have been no extreme changes in land cover before, during, and after the COVID-19 pandemic. This condition has allowed us to ascertain the socio-managerial dynamics among managers and migrant recipients of customary forest and village forest land (see Figure 1).

Tenganan Village is located in Manggis District, Karangasem Regency. The population is 1,044 people. The people of Tenganan Village are an early Hindu community (Bali Aga) with the Indra sect. They do not recognize caste systems like those found among Balinese people in general. Based on Decree number 1546/MenLHK-PSKL/PKTH/Kum.1/2/2019, the Minister of Environment and Forestry (MoEF) designated the forest in Tenganan as a customary forest. The Regulation of the MoEF Number 9 of 2021 concerning social forestry management stated that customary forests are located within the territory of customary law communities. Customary areas refer to lands and/or waters along with the natural resources thereon, that have certain boundaries, and are owned, utilized preserved, and sustained from generation to generation to meet the needs of the community. The customary lands or forests are inherited from ancestors or acquired through the claim of ownership. The area of the Tenganan customary forest extends approximately 591 hectares consisting of 226 hectares of protected forests and 365 hectares of productive forests. The Tenganan customary forest is managed by all indigenous peoples, numbering around 668 people or 225 families. All residents are Hindus ( BPS 2020). They are guided by customary rules ( *awig-awig *) in managing customary forests.

Wanagiri Village is situated in Sukasada District, Buleleng Regency. It has village forest that managed by a Village-Owned Enterprise named “Eka Giri Karya Utama”. With a total of 250 hectares, this village forest is divided into 2 zones of 80 hectare of protection zone and 170 hectare of utilization zone. The village forest was designated with the Decree of the Governor of Bali Number 2017/03-L/HK/2005. The Village forest is a forest areas that have not been burdened with permits. The village forests are customarily managed and utilized by the village for the welfare of the village, village areas, or areas resulting from management boundary agreements between adjacent villages. The community maps them in a participatory manner, and/or located within a single natural landscape in the village. Most of the village forests have been planted with coffee. The total population in the village of Wanagiri is 4,056 people; 51.6% of which are men and 48.4% are women. Religion of the population are Hindus (98.9%); Islam (0.7%); Christian (0.2%); Catholic (0.1%); and Budhis (0.1%) ( Sistem_Informasi_Desa, 2023). There are 296 families involved in village forest management. This number is divided into three forest farmer groups, namely Wana Amerta (with 78 families); Puncak Manik (35 families); and Jagra Wana (78 families).

In order to protect the public’s health during the COVID 19 epidemic, the government imposed travel restrictions, promoted the 3M campaigns (Memakai masker, Mencuci tangan, dan Menjaga jarak/mask use, hand washing, and maintaining social distancing), and administered vaccinations. In addition, the government also ensured the digitalization of health care. When a positive case of COVID-19 is suspected, medicine is immediately sent free of charge. Not all countries allow free transport of medically prescribed medicine.

**Figure 2.**
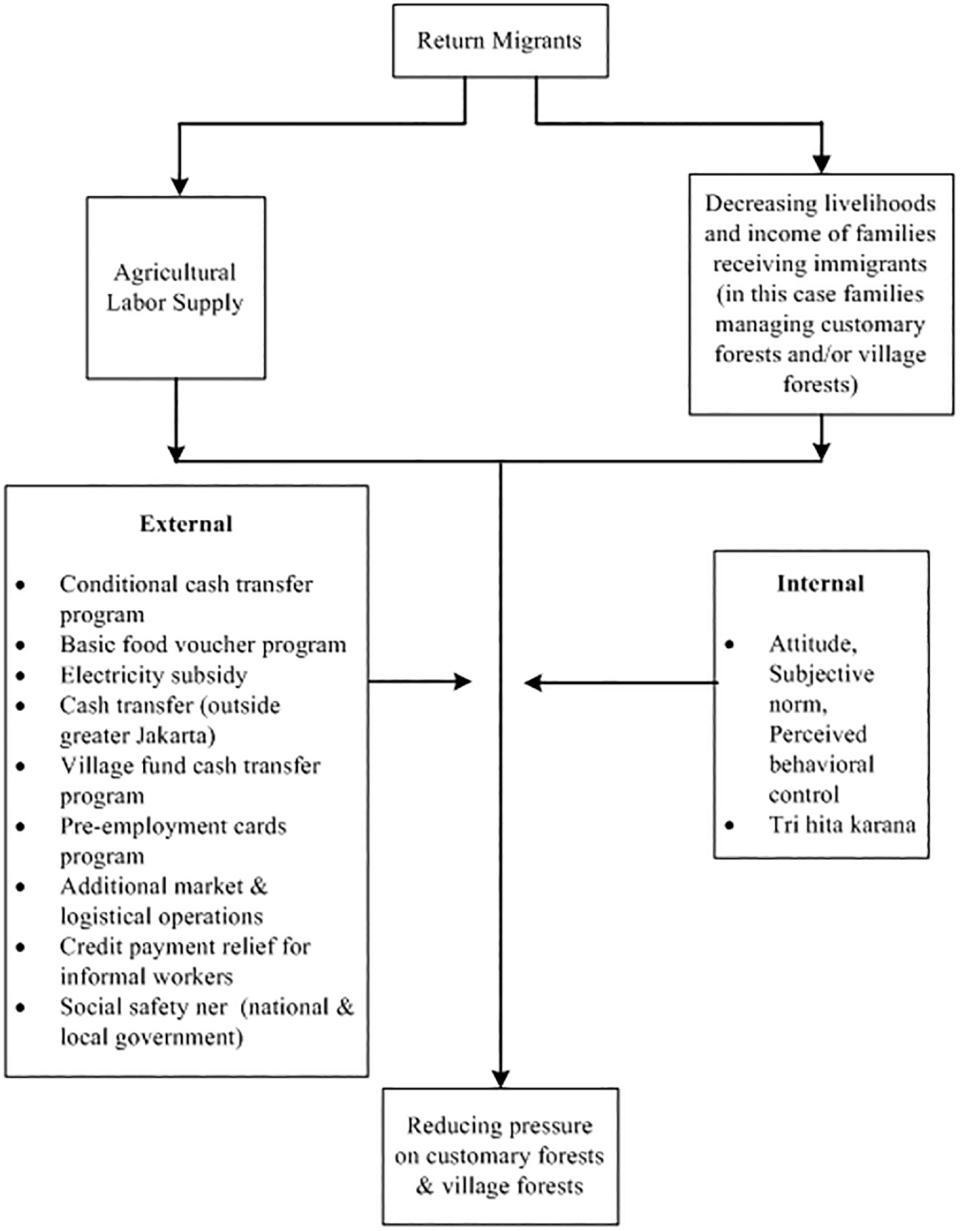
Framework return migrants and forest relations in the pandemic. Note: Figure 2 modified from Bista et al. (2022).

From an economic standpoint, the Government implemented a partial lockdown or locally known as Large-Scale social restriction (PSBB). This is quite rational because people can still carry out economic activities. The enforced PSBB in these areas is considered far more realistic than implementing a complete lockdown national wide ( Roziqin et al. 2021). In addition, the government also provides a social safety net. There are several social policies which include Family Hope Program, Staple Food Cards, Pre-Employment Cards, electricity subsidies, additional market and logistics operations, relief of credit payments for informal workers, and BLT Dana Desa (direct cash assistance to the village) (for more details, see Figure 2).

**Figure 3.**
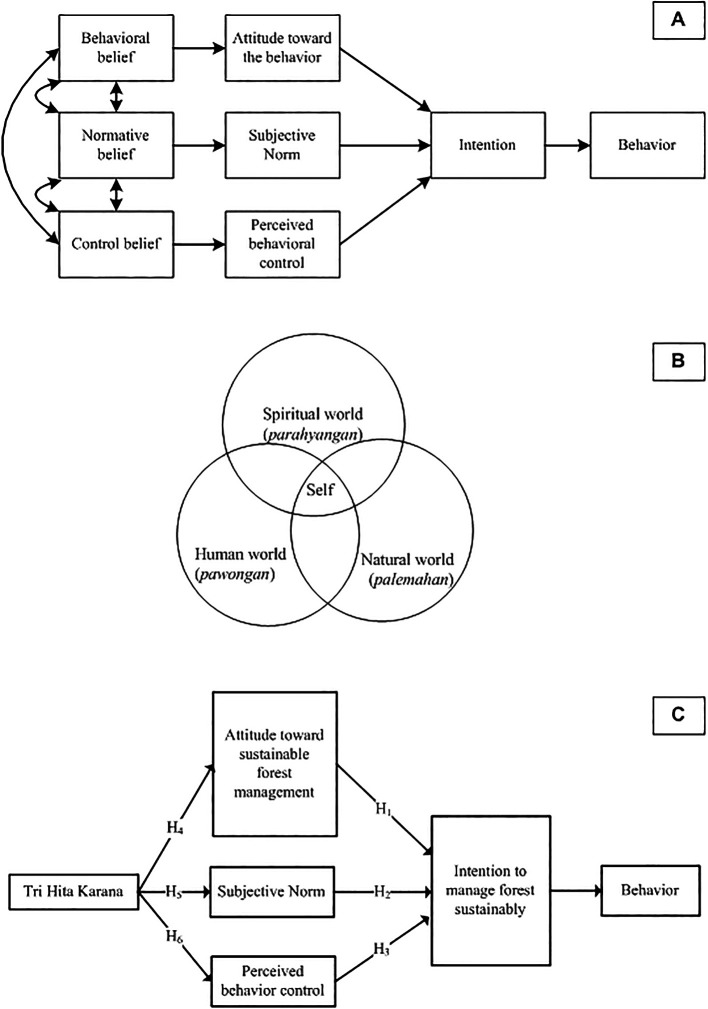
Planned Behavior Theory, Tri Hita Karana, Proposed model and hypotheses. Note: Planned Behavior Theory (A). Source: Yuriev et al. (2020); Tri Hita Karana (B). Source: Adityanandana and Gerber (2019).

Operationally, the research question of this study can be illustrated in Figure 3.

As seen in Figure 3(A), attitudes, SN, and PBC are the predictors of intention. Consequently, this study examines three hypotheses derived from the conceptual model:

**Table:** 

*H _1_:*	Attitudes have a positive effect on the intention to manage forest sustainably
*H _2_:*	Subjective norm has a positive effect on the intention to manage forest sustainably
*H _3_:*	Perceived behavioral control has a positive effect on the intention to manage village and/or customary forest sustainably

As with the general application of TPB theory, individual serves as the unit of analysis in this study. THK is defined as the level of respondent's internalization of the Human-God, Humans-Humans, and Humans-Nature dimensions, as measured with the respondent's (individual's) subjective perception. Therefore, THK has different values ​​for different individuals. Figure 3(B) illustrates that THK represents a harmonious integration of three related realms: the human world (pawongan), the natural world (palemahan), and the spiritual world (parahyangan). The self (microcosm) is indispensable from the universe (macrocosm) and both are composed of the same elements ( Adityanandana and Gerber 2019). Furthermore, Citing Flood (1997) and Agung (2005), they explained that in THK, violence against nature is self-harming. Nature is believed to be a manifestation of the Supreme Being, and accordingly, is fundamentally sacred. Nature is not a merely human-exploited object, but instead, it deserves respect, as captured in the section Svet a svataropanisad: Submit to God because God is in the fire, in the air, in the entire universe, in the plants above the trees”. The THK concept of the spiritual world is relevant to the doctrine of entelechy. This doctrine is classically outlined in Purusartha (the object of human pursuit), which consists of moral (dharma), economic (artha), sensual (kama), and spiritual (moksha) values. In spite of their whole importance, the pursuit of economic and sensual desires should not override moral values ​​in order to achieve spiritual fulfillment (the ultimate goal—moksha). The path to moksha (or liberation from suffering, inherent in the cycle of rebirth) unfolds over several lifetimes depending on one's actions (karma) and the soul's maturity to detach itself from the physical world.

The framing of *THK* as “culture”, “tradition”, and “local wisdom” can be criticized by using insights from various scientific domains ( Roth and Sedana 2015). In this study, we frame *THK* as a value. Value is a belief that is closely related to influence. When values are activated, they are infused with feelings ( Schwartz 2012). He also gives an example of people who consider independence an important value. People become aroused when their independence is threatened. They may feel despair when they are powerless to protect it and feel happy when they can enjoy it. Thus, the hypothesis proposed is:

**Table:** 

*H _4_:*	*THK* has a positive effect on attitudes towards sustainable forest management
*H _5_:*	*THK* has a positive effect on subjunctive norms
*H _6_:*	*THK* has a positive effect on perceived behavioral control

Finally, Figure 3(C) shows the model proposed by this study, where value (THK) is an antecedent of TPB.

### Sample

Purposive sampling was used in this study. According to Cooper and Schindler (2013), purposive sampling is a non-probabilistic sampling that meets specific criteria. Following of the study objectives, the specific criteria are people who: (1) cultivators of customary forests and village forests; (2) adults; (3) have a good literacy level; (4) responsible for the laid-off immigrants due to the COVID-19 pandemic; and (5) willing to be involved in the study.

The sample size for a multivariate analysis should be 10 times more than the total number of variables to be examined, according to Roscoe in Sekaran and Bougie (2016). Depending on the complexity of the model, a sample size of 5 or 10 or 15 cases per parameter ( Kline 2016). Meanwhile, the number of representative samples used in multivariate analysis was between 100 and 200, or five times as many as the questionnaire’s question items ( Hair et al. 2018). Based on what has been stated and also because not all managers of customary forests and/or village forests accept migrants, we targeted a sample size of 200 respondents.

### Measurement

In this study, structured questionnaires were used. The questionnaire consisted of two major parts, namely: (1) inquires about *THK*, attitudes, SN, PBC, and intention to continue to manage forest sustainably; and (2) inquires about the respondent’s profile. The question items were modified from earlier studies by Ariyanto et al. (2017); Homer (1995); Ofoegbu and Speranza (2017); Buyinza et al. (2020); Borges and Lansink (2016). Because the question items are translated from English to Indonesian, the accuracy of the translation matter ( Jogiyato 2013). Therefore, we asked linguists at Yogyakarta State University to translate the question items from English into Indonesian. The translated Indonesian version was translated back into English by the author’s colleagues who had studied abroad to identify any significant differences.

### Data analysis

The data in this study were analyzed using SEM. There are two SEM methods: covariance-based (CB-SEM) and variant-based (PLS-SEM). When deciding which one to be utilized, it’s critical to be aware of the differences between the two ( Hair Jr. et al. 2017). CB-SEM aims to “minimize the differences between sample covariance matrix estimates, while PLS-SEM maximize the explained variance of endogenous constructs” ( Hair et al. 2011). Therefore, CB-SEM is mainly used for the confirmation of established theories (explanations); in contrast, PLS-SEM is a prediction-oriented approach, primarily undertaken for exploratory research ( Sarstedt et al. 2014).

Almost all studies using PLS-SEM state that PLS-SEM has advantages over CB-SEM. It can complete formative and reflective measurements. Another advantage is that the samples are not necessarily large. Besides that, it assumes that the samples are not necessarily normally distributed ( Hair et al. 2014; Henseler et al. 2009). Because one of the aims of this study is to predict whether *THK* is an antecedent of attitude, SN, and PBC, this study uses PLS-SEM.

Although PLS_SEM has some advantages, it also has disadvantages. PLS-SEM does not have a Goodness-of-Fit (GoF) index. The geometric mean of the communal mean and average R2 can be used as general criteria for GoF ( Tenenhaus et al. 2005). The criteria for small, medium, and large effects of GoF are 0.1, 0.25, and 0.36 ( Wetzels et al. 2009).

## Results

### Profile of respondent

The respondents were surveyed using a self-administered questionnaire. To filter respondents to fit the criteria, we used filter/screening questions. The questions asked whether, during the COVID-19 pandemic, the respondents accepted the laid-off family members. Screener questions were intended to avoid respondents from answering irrelevant questions.

Of the 200 questionnaires distributed, 71 respondents completed them (met the criteria sample and pass the screener question). Therefore 71 questionnaires were analyzed. Of the 71 data analyzed, the respondents were born and raised in the villages of Wanagiri and Tengganan (97.2%) while the rest were not born in the location and live in the villages of Wanagiri and Tengganan due to marriage (2.8%). Table 1 contains information about the respondent’s profile.

**Table 1.** Respondent’s demographic profile (n = 71).

**Gender**		**Monthly expenses**	
Male	78.90	≤ Rp 1.000.000	12.70
Female	21.10	Rp. 1.000.001 – Rp. 2.500.000	64.80
		> Rp. 2.500.000	22.50
**Age**		**Occupation**	
< 21	2.80	Farmer	59.20
21-30	8.50	Village officials	8.50
31-40	22.50	Trader	11.30
41-50	35.20	Other	21.10
> 50	31.00		
**Education**		**Family members**	
Elementary School	35.20	< 2	15.5
Junior High School	19.70	2-4	63.4
Senior High School	25.40	> 4	21.1
Academy	19.70		

### Measurement model

A self-administered version of the questionnaire was intended. When employing self-administered questionnaires, researchers encounter challenges since respondents’ answers are more impacted by the clarity of the written words than by the interviewer’s abilities ( Zikmund and Babin 2016). Thus, the initial step was to carry out a pilot test after the questionnaire had been compiled. The objectives of the pilot test are to identify: (1) whether there are ambiguous words; (2) whether the instructions given can be understood; (3) whether it is difficult for the respondent to answer; and (4) how long the respondent took the time to fill out the questionnaires. A tiny sample size of three respondents participated in this pilot test. The questionnaires were promptly duplicated and distributed to the respondents after revisions were made in response to the pilot test’s findings.

The criteria for convergent validity, according to Fornell and Larcker (1981), are that: (1) the factor loading is significant and higher than 0.7; and (2) the Average Variance Extracted (AVE) value is higher than 0.5; whereas for discriminant validity, the AVE value exceeds the squared correlation value between the construct pairs ( Table 3). The composite reliability value is used to evaluate reliability. The cutoff criterion for Composite Reliability is 0.7 ( Abdillah and Jogiyanto 2015; Nunnaly in Onofrei et al. 2022).

Table 2 shows that all variables passed the convergent validity test with an AVE value greater than 0.5. Additionally, it has passed the test for discriminant validity, which establishes that each indicator in a latent variable differs from indicators in other latent variables (as shown by a higher loading score in its construct). All variables pass the construct reliability test, as evidenced by the reliability testing results (each variable’s composite reliability is more than 0.7).

**Table 2.** Convergent validity and reliability testing.

Constructs	Item loading	AVE	Composite reliability
***Tri Hita Karana*** ( Ariyanto et al. 2017)		**0.690**	**0.917**
• Sincerity and prayer will expedite my process of utilizing forest land	0.766		
• Believing in the law of karma phala will lead me to the forest land utilization	0.854		
• Village (customary) leaders care for forest land utilization	0.851		
• Collective effort and responsibilities of village (customary) residents and leaders ensure the wise utilization of the forest	0.783		
• Forest land utilization provides learning opportunities and enables anticipation of upcoming changes	0.894		
**Attitude toward SFM** ( Homer 1995)		**0.851**	**0.945**
Sustainable management of customary/village forests to reduce the economic burden after the pandemic is:			
• Very Unwise – Very Wise	0.941		
• Negative- Positive	0.857		
• Very Poor – Very Good	0.966		
**Subjective Norm** ( Ofoegbu and Speranza 2017)		**0.679**	**0.863**
• Village and/or customary officials will support the sustainable management of customary forests/village forests	0.761		
• Other people with whom I interact regularly will perceive the desirability of involvement in the sustainable management of customary village forests	0.835		
• I appreciate other people's significant opinions regarding my involvement in the sustainable management of customary/village forests	0.872		
**Perceived Behavioral Control** ( Buyinza et al. 2020)		**0.693**	**0.871**
• I believe I am knowledgeable enough about sustainable customary/village forest management	0.759		
• I have all the necessary labor and knowledge resources to manage village and/or customary forests sustainably	0.874		
• When I want to plan sustainable customary/village forest management, I have sufficient technical skills	0.860		
**Intention toward SFM** ( Borges and Lansink 2016)		**0.862**	**0.926**
• I intent to get alternative income from sustainable customary/village forests management in the next year?	0.898		
• How serious are you in the customary/village forests management in the next year?	0.921		

**Table 3.** Discriminant validity testing.

	Attitude toward SFM	Intention toward SFM	PBC	Subjective norm	Tri Hita Karana
Attitude toward SFM	**(0.922)**				
Intention toward SFM	0.401	**(0.928)**			
PBC	0.233	0.428	**(0.832)**		
Subjective Norm	0.470	0.471	0.365	**(0.824)**	
Tri Hita Karana	0.292	0.352	0.372	0.312	**(0.831)**

Note: The values in brackets are the square root of AVE.

### Structural model, hypothesis testing and goodness of fit

Following the measurement model, SEM was used to investigate each hypothesis contained within the suggested model. This two-step analytic strategy is consistent with Anderson and Gerbing (1988). The results can be seen in Table 4.

**Table 4.** Structural model and hypotheses testing.

Hypotheses		Path Coefficient	*t-value *	Supported?
*H _1_*	Attitude → intention	0.112	2.063	Yes
*H _2_*	Subjective norm → intention	0.130	2.539	Yes
*H _3_*	Perceived behavioral control → intention	0.111	3.292	Yes
*H _4_*	*Tri Hita Karana* → atiitude	0.121	3.471	Yes
*H _5_*	*Tri Hita Karana* → subjective norm	0.148	3.029	Yes
*H _6_*	*Tri Hita Karana* → perceived behavioral control	0.092	5.841	Yes

Table 4 shows that all hypotheses are supported by data with a t value greater than the t table; while the relationship between variables shows unilateral results (all path values have positive coefficients).

Before testing the hypothesis, we calculate the Goodness-of-Fit (GoF). Overall, GoF should be the starting point for model assessment. If the model does not fit the data, then the data contains more information than the model can convey ( Henseler et al. 2016). The benefits of GoF are: (1) Assessing Model Predictive Ability. The main benefit of GoF is that it is a single index to evaluate the overall quality of both measurement models and structural models ( Tenenhaus et al. 2005); (2) Preventing Information Failure. GoF serves to ensure that the model does not “throw away” important information contained in the data. Lack of fitness of the model will result in debatable conclusions ( Henseler et al. 2016), (3) Providing Legitimacy to the Model (Parsimony). GoF is beneficial to provide evidence the simple model can be very effective in explaining complex phenomena ( Wetzels et al. 2009). The GoF value in this study is 0.685. Referring to Henseler et al.’s (2016) criteria, this value exceeds the threshold for the large category, indicating that the proposed model has high consistency with the data. Therefore, the proposed model is consistent with the data, and the tested model is parsimonious and reasonable.

## Discussion

Social forestry often fails to provide full rights to local communities, especially in the context of customary rights and formal recognition ( Wong et al. 2020). This is particularly true because participation is limited to village elites and technical support is almost non-existent. In other words, the main obstacles include bureaucratic processes and local actor exclusion ( Royer et al. 2018). However, Decree number 1546/MenLHK-PSKL/PKTHA/Kum.1/2/2019 of the Minister of Environment and Forestry (KLHK) that determined the forest in Tenganan as a customary forest; Decree of the Governor of Bali Number 2017/03-L/HK/2005 that determined it as a Village Forest in Wanagiri; and Decree of the Governor of Bali Number 2017/03-L/HK/2015 as well as Decree of the Buleleng Regent Number 430/405/HK/2017 concerning the Management of Tourism Villages in Wanagiri, have reduced some of these detrimental factors. This aligns with social forestry’s key success factors ( Gilmour 2016): secure tenure rights, supportive regulation, strong governance, market access, and bureaucratic support.

The residents of Wanagiri Village have established a tourism awareness group as a part of the village-owned enterprise (BUMDes). Their income is derived from managing local natural tourist attractions such as Banyumala Waterfall, Puncak Manik Waterfall, and Buana Sari Waterfall, as well as providing tour guides ( Laksemi et al. 2019). Local entrepreneurship practices in Wanagiri Village are organized by the BUMDes “Eka Giri Karya Utama”. The economic programs include savings and loans, tourism, coffee processing, waste management, and water management. They also developed LPD (Village Credit Institution) a village-level credit institution to provide financial access.

Likewise, the residents of Tenganan Village maintain the customary system and local wisdom in forest management. The community applies awig-awig to maintain and sustain the forest, even before receiving formal recognition from the government. These awig-awig are customary regulations that contain recommendations and prohibitions (for example, the prohibition of cutting down trees and changing the function of forest land without permission from the Traditional Village). The highly obedient community reflects the effectiveness of the awig-awig. Most resident whose lives are highly dependent on the sale of commodities, especially palm leaf crafts, Gringsing cloth (whose materials are collected from customary forests) stated that the tourism industry in Tenganan Village is very important and needs to be continuously developed. However, some indigenous people perceive tourism development as a mere bonus of the strengthening of local culture.

The two cases showed that SF in Wanagiri is more proactive in articulating ideas and social participation in village deliberation forums, while SF in Tenganan has provided full rights to the local community.

When the COVID-19 pandemic first occurred in Italy, demand for wood contracted. The international market (export-import) of wood products experienced a sharp decline ( Barcaccia et al. 2020). Likewise in China, the price of natural resource commodities is volatile and short termed due to the disrupted supply and demand chains resulting from the increase in active cases and the spike in deaths of COVID-19 patients ( Guo et al. 2022). For the case in Indonesia, there was a decrease in income in the wood processing industry business in Labe Lawe, Sekadau Hilir Regency, West Kalimantan, from previously IDR 492,927,000 before the Covid-19 pandemic and IDR 345,583,000 during the Covid-19 pandemic. It implies an income discrepancy of IDR 147,344,000 or a decrease of up to 30.0% ( Widhanarto et al. 2024). By comparing the impact of COVID-19 to that of previous economic crises ( Wunder et al. 2021), found that national income and commodity prices are affected by 3 factors, namely: contractionary-inflationary supply-side shocks, deflationary demand-side impacts, and expansionary-inflationary government policy responses (both monetary and fiscal). In the US, the COVID-19 pandemic caused a significant shift in the demand function, while the supply function shifted inward due to labor shortages. The lumber price in the US increased from $319.70 per thousand board feet (mbf) in April 2020 due to the appearing sign of pandemic resolution. It reached the peak of $1500.50/mbf exactly one year later ( van Kooten and Schmitz 2022).

Literatures reveales that deforestation (pressure on forests) has resulted from the increased conversion of forest land when compared to the desire to control the trees ( Kaimowitz and Angelsen 1998; Angelsen and Kaimowitz 1999). In general, this conversion aims at opening up land for industry, settlements, plantations, agriculture, mining and others. When the COVID-19 pandemic occurred, this pressure increased with layoffs and return migration. When layoffs hit, they generally return to their hometowns to seek new opportunities, find temporary housing, and/or look for emotional and social support. On the other hand, their hometowns offer very few formal employment opportunities that match the skills of the return migrants. Reduced or without income will force the return-migrant to turn to natural (agricultural) resources for survival. This will subsequently put pressure on the existing agricultural land, which in turn will encourage return-migrant to engage in activities that exploit natural resources including forests.

The results of this study differ from the findings of research conducted by Yazdanpanah et al. (2014) who examined water conservation-related behavior intentions across the Middle East and North Africa; Knussen et al. (2004) who examined intention to recycle household waste in Glasgow, Scotland; Ofoegbu and Speranza (2017) who examine at South Africa’s intention to adopt practical management and sustainable forest usage; where the three reported that at least one of the 3 predictors of behavioral intention in TPB (Attitude, SN and PBC) did not have a significant effect. Accordingly, the results of this study confirmed that the 3 predictors had a positive and significant effect. The findings of this study are consistent with the study conducted by Ajzen (2011) who states that ideally, the 3 predictors have a positive and significant statistical effect; Borges and Lansink (2016) who predicted cattle ranchers’ intentions in Brazil to adopt better natural pastures.

This study used the TPB model to understand, explain, and predict the behavior of families who receive the arrival of migrants in their areas. It identifies the reason for their willingness to cultivate forests sustainably. This study model includes the *THK* construct into the TPB model to understand, explain, and predict the behavior of land cultivators due to the impact of COVID-19. When COVID-19 hit, many company workers were laid off so they returned to their hometowns, which consequently more or less put pressure on the families who received their return.

The TPB model developed in this study shows that social psychological factors (attitudes, SN, and PBC) can explain and predict the intentions and behavior of forest managers. The *THK* variable included in the TPB model can explain and predict attitudes, SN, and PBC positively and significantly. Adopted values are defined as ideals and guiding principles in human life ( Rokeach 1973; Schwartz 1992). Likewise with *THK* are the values adhered to by the Balinese Hindu community and become the basis for displaying behavior.

Attitudes have a strong impact on people’s perceptions toward the attitude object and thus have an impact on behavior ( Fazio 1986). Attitudes can be positive or negative and contain moral beliefs, namely individual beliefs that something is moral or immoral ( Eagly and Chaiken 1993; Krosnick and Petty 1995; Skitka et al. 2005). Attitudes have a strong influence on the way humans perceive and understand the world ( Fazio 2000; Maio et al. 2019).

Because values are guiding principles and they are considered to guide our behavior ( Sagiv and Roccas 2017) through a series of variables including attitudes ( Homer and Kahle 1988), then, the values espoused will influence human feelings towards certain objects or people, which in turn will influence action ( Thorne et al. 2020). In this study, the values of *THK* adhered to by migrant-receiving families can shape their attitudes toward sustainable forest management. They adhere to the values of *THK* principles that produce positive manifestations reflected in the continuous management forests sustainably even though they have migrants arriving.

Furthermore, espoused values may vary between individuals depending on their personality, needs, and circumstances ( Sheth et al. 1991). Adhered values are felt by individuals and can be shaped and influenced by others as many research results show that individuals are influenced by friends, relatives, co-workers, business partners, or other parties around them ( Paul et al. 2016). The results of this study indicate that the perception of migrant-arriving families to *THK* values is positive. This in turn forms the SN. The family feels that the *THK* values around them can influence their perception of SN. In this case, the reference group agrees or advises them to continuously carry out sustainable forest management. In other words, the reference group also adheres to *THK* values and may also sustainably manage forests.

PBC refers to a person’s beliefs about how easy or difficult or possible or impossible it is to perform a particular behavior ( Ajzen 1991). Many previous studies often used PBC as an antecedent of various behaviors related to environmental sustainability ( Fishbein and Ajzen 2010; Yuriev et al. 2020). PBC contains belief power which is an individual’s belief in the existence of factors that support him to behave. This belief is a consequence of the values held by the individual. The *THK* values adhered to by migrant-receiving families control their behavior concerning sustainable forest management. The espoused value is an individual’s belief in the existence of factors that support him to manage forests sustainably. Thus, the espoused *THK* values can influence attitudes, SN, and PBC, which in turn influence the intention of cultivating forest land sustainably.

### Conclusions

This study reveals how the COVID-19 pandemic has not had a significant impact on forest management specially on Tenganan forests and Wanagiri forests in Bali. This study has presented an enhanced version of the TPB model that adds a new variable called THK to explain the behavior of migrant-receiving families in order to provide an answer to the condition. The conclusion is that THK, which is a value in Balinese society, is an antecedent predictor of behavioral intention to manage forests sustainably, mediated by attitude, SN, and PBC.

The effect of THK on behavioral intentions is mediated by subjective norms. This suggests that THK functions as a collective moral compass rather than merely an individual belief. In the Balinese context, the THK philosophy is embedded in social expectations. Therefore, individuals are socially enforced to practice sustainable forest management by maintaining harmony with their community (Pawongan) and their environment (Palemahan), as expected by their social environment.

THK significantly affects behavioral intentions through the mediation of perceived behavioral control. This suggests that THK values — particularly social cohesion found in Pawongan (formal rule, customary law, or economic necessity)—strengthen individuals' sense of independence and self-efficacy. THK has improved respondents’ sense of empowerment and perceptually reduced barriers to practice sustainable forest management, as they relied on the collective strength and traditional institutional support inherent in the THK framework.

### Limitations

This study has several limitations for further research. **First**, the data was collected using the cross-sectional method that captures only a specific point in time. Further research may consider the longitudinal method. **Second**, the non-probabilistic sampling strategy is applied in this study to acknowledge several limitations to the external validity of the findings. Rather than offering universal generalizations across all social forestry models, the results of this study apply only to villages with similar socio-ecological profiles. In particular, these findings represent autonomously and traditionally managed by villages based on customary law (awig-awig), with communal land ownership, and the state-owned forests managed collaboratively by the Village Forest Management Institution and the government, namely the Ministry of Environment and Forestry. **Third**, in Government Regulation Number 23 of 2021, social forestry has five schemes namely village forests, community forests, community plantation forests, customary forests, and forestry partnerships. This study was exclusively conducted in village forests and customary forest, and future research could be conducted on other schemes of social forestry.

This study also has some practical implications for managers. Because the *THK* concept can guide humans in humanizing nature by harmonizing the concepts of God and humans, by socializing, understanding, deepening, and applying the *THK* concept, awareness will be created to protect nature because nature is part of human beings and God. In other words, in creating community welfare, there is an inseparable relationship between humans and God. A deeper understanding and application of this approach can be ensured by making *THK* a mandatory content and subject in the primary and secondary education curriculum.

### Ethical considerations

This study received ethical approval from the Ethics Committee of the Indonesia Open University (Universitas Terbuka Indonesia) following comprehensive review (Protocol Number: B/1571/UN31SPS/PT.01.05/2024, approved on April 19,2024). Due to educational background, cultural norms, and risk perception, verbally informed consent was obtained from all participants prior to their involvement in the study. Participants provided explicit agreement for their response to be published in anonymized form as part of aggregate data analysis. All data collection and management procedures adhered to established ethical guidelines for human subject research. To improve the wording and readability of this study, the authors used Grammarly.

## Data Availability

Figshare: Dataset coding responses from Bali-THK respondents (English).xlsx, 10.6084/m9.figshare.27890262.v1 ( Patiro et al. 2024). This file contains the following underlying data:
* Dataset coding responses from Bali-THK respondents (English).xlsx * The variable in this file are: Name, Full-time job, Part-time job, Gender, Length of stay, Monthly expenditure, Age, Education, Family members, Social position, Marital status, Tri Hita Karana (5 indicators), Attitude (3 indicators), Social Norm (3 indicators), Perceived Behavioral Control (3 indicators), and Intention (2 indicators). Data are available under the terms of the Creative Commons Zero “No rights reserved” data waiver (CC0 Public domain dedication). 1. Figshare: Tri Hita Karana Questionnaire (Indonesia).docx, 10.6084/m9.figshare.27861828.v1 ( Patiro et al. 2024). This file is a questionnaire in Bahasa Indonesia.
2. Figshare: Tri Hita Karana Questionnaire (English).docx, 10.6084/m9.figshare.27861894.v1 ( Patiro et al. 2024). This file is a questionnaire in English. Data are available under the terms of the Creative Commons Zero “No rights reserved” data waiver (CC0 Public domain dedication). This work did not use standard review methods, hence no reporting guidelines were applied.
