# Supplementary material for: An extended Theory of Planned Behavior in explaining intention toward sustainable forest management: Evidence from COVID 19 Pandemic from Bali, Indonesia
Source: F1000Res. 2026 Apr 6;13:1457. Originally published 2024 Dec 2. [Version 4] doi: 10.12688/f1000research.158455.4 (PMC12831049; doi:10.12688/f1000research.158455.4)
Supplement: Peer review history

# Reviewer report: Reviewer response for version 3

**DOI:** 10.5256/f1000research.184518.r451863

**Published:** 2026-01-23

**Authors:** I Gusti Putu Bagus Sasrawan Mananda

**Affiliations:** 1 Udayana Universty, Bali, Indonesia

Summary of the Article

 This article examines intentions toward sustainable forest management among customary and village forest managers in Bali during the COVID-19 pandemic using an extended Theory of Planned Behavior (TPB). The authors integrate *Tri Hita Karana* (THK), a core Balinese value system, as a value-based antecedent influencing attitudes, subjective norms, and perceived behavioral control, which in turn shape behavioral intentions. Primary data were collected from 71 forest managers in two villages using structured questionnaires, and the proposed model was tested using Partial Least Squares Structural Equation Modeling (PLS-SEM). The results show that all TPB components significantly predict intention, and that THK significantly influences each TPB component. The authors conclude that culturally embedded values play a critical role in shaping pro-environmental intentions, especially under crisis conditions such as the COVID-19 pandemic. Overall, the study makes a meaningful theoretical contribution by extending TPB with a culturally grounded value construct and provides relevant insights for social forestry policy and practice in Indonesia.

 Clarity of Presentation and Use of Literature

 Assessment: Yes

 The manuscript is clearly written, well structured, and logically organized from introduction through conclusions. The research objectives are explicitly stated, and the conceptual framework is coherently developed. The authors demonstrate strong engagement with both foundational literature (e.g., Ajzen, Schwartz, Stern) and recent, relevant studies related to COVID-19, environmental behavior, and social forestry, including sources published up to 2024–2025. The revisions across versions have improved clarity, terminology consistency, and contextual depth. Citations are appropriate, comprehensive, and accurately integrated. No major issues are identified in this area.

 Study Design and Technical Soundness

 Assessment: Partly

 Although the overall study design is appropriate for the stated aims and the use of an extended TPB framework in a culturally specific and crisis-affected context is theoretically justified, several data-related and design-related questions must be addressed through revision of the manuscript.

1. First, the manuscript must explicitly justify why only two villages were selected as the empirical basis for the study, given that Bali contains many forested villages actively involved in forest management. The authors need to revise the methods section to answer the following questions: Why were Tenganan and Wanagiri chosen over other villages managing customary or village forests? Are these villages intended to represent typical, exemplary, or theoretically extreme cases of forest governance in Bali? On what empirical or theoretical grounds can behavioral intentions observed in only two villages be considered informative for understanding sustainable forest management intentions in Bali more broadly? Without a clear and explicit justification, the restricted spatial scope raises concerns about selection bias and limits interpretability.
2. Second, the authors must revise the manuscript to explain how Tri Hita Karana operates analytically as a variable rather than as a cultural constant. Tri Hita Karana is a foundational Balinese philosophy emphasizing harmony in Parahyangan (humans–God), Pawongan (humans–humans), and Palemahan (humans–nature). Given that THK values are widely shared across Balinese society, the authors need to address the following question: What meaningful variation in THK-related values exists between respondents, villages, or forest governance systems that allows THK to explain differences in behavioral intention? Without addressing this question, it remains unclear whether the observed effects reflect genuine value-driven behavior or the influence of deeply institutionalized norms that are common across Bali.
3. Third, the manuscript must address whether the behavioral intentions measured are driven by THK values per se or by institutional roles and obligations associated with being a forest manager. The authors should revise the interpretation of results to answer the following question: To what extent do respondents act sustainably because of internalized THK values (spiritual, social, ecological harmony), and to what extent because of formal rules, customary law, or economic necessity during the COVID-19 period? This distinction is essential to avoid conflating cultural values with governance compliance.
4. Fourth, the adequacy of the final sample size of 71 respondents must be more rigorously justified within a PLS-SEM framework. While PLS-SEM can accommodate smaller samples, the authors should revise the methodology section to answer the following question: How does the achieved sample size meet accepted minimum sample size or statistical power criteria for the proposed model (for example, using inverse square root or gamma-exponential methods)? Reliance on general heuristics alone is insufficient for establishing model stability.
5. Fifth, the manuscript must more clearly revise its claims to address the implications of purposive, non-probability sampling. Specifically, the authors should answer the following question: How do the sampling strategy and village selection constrain the external validity of the findings, and to which types of villages or forest governance contexts can the results reasonably be applied? This revision is necessary to prevent implicit generalization beyond comparable social forestry settings.

Methods and Replicability

 Assessment: Yes

 The methods section provides sufficient detail to allow replication. The authors clearly describe the study context, participant criteria, measurement instruments, translation and back-translation procedures, and data analysis steps. Construct sources are properly cited, and validity and reliability assessments are reported transparently. Importantly, the authors make the full dataset, coding file, and questionnaires publicly available through Figshare under a CC0 license. This significantly enhances transparency and reproducibility and meets best practices in open science. One minor issue that does not undermine replicability but merits clarification is the pilot test, which involved only three respondents. While acceptable as a basic face-validity check, the authors should clarify that this pilot was used solely for wording and comprehension rather than psychometric validation.

 Statistical Analysis and Interpretation

 Assessment: Partly

 The statistical analysis is largely appropriate and competently executed. The authors correctly assess convergent validity, discriminant validity, and composite reliability. Structural paths are interpreted consistently with TPB theory, and the reported results align with the stated hypotheses. However, there are two methodological points that should be addressed to strengthen rigor. First, although the authors report a Goodness-of-Fit (GoF) index, the use of GoF in PLS-SEM remains debated in the literature. The manuscript should acknowledge this limitation more explicitly and, if possible, supplement the evaluation with additional recommended indicators such as effect sizes (f²), predictive relevance (Q²), or out-of-sample prediction metrics. Second, the study interprets statistically significant paths without sufficient discussion of their relatively small effect sizes. While significance is achieved, the practical magnitude of some coefficients appears modest. The authors should briefly discuss this to avoid overstating the strength of relationships.

 These revisions are analytical and interpretive rather than structural and can be addressed within the existing results section.

 Availability of Source Data

 Assessment: Yes

 All underlying data, extended data, and instruments are openly available and clearly documented. The data availability statement is transparent, and the licensing allows full reuse. This aspect of the manuscript is exemplary and requires no changes.

 Support for Conclusions

 Assessment: Yes

 The conclusions are well supported by the results and remain appropriately cautious. The authors do not claim direct behavioral outcomes beyond intention and clearly acknowledge the limitations related to cross-sectional design, sampling, and scope. The theoretical implications regarding value-based extensions of TPB are justified by the findings, and the practical recommendations are aligned with the cultural and institutional context studied.

Is the work clearly and accurately presented and does it cite the current literature?

Yes

If applicable, is the statistical analysis and its interpretation appropriate?

Partly

Are all the source data underlying the results available to ensure full reproducibility?

Yes

Is the study design appropriate and is the work technically sound?

Partly

Are the conclusions drawn adequately supported by the results?

Yes

Are sufficient details of methods and analysis provided to allow replication by others?

Yes

Reviewer Expertise:

Tourism, overtourism, behaviour

I confirm that I have read this submission and believe that I have an appropriate level of expertise to confirm that it is of an acceptable scientific standard, however I have significant reservations, as outlined above.

# Response

**Published:** 2026-03-21

**Authors:** Kresno Agus Hendarto

**Affiliations:** Pusat Riset Kependudukan, Badan Riset dan Inovasi Nasional Republik Indonesia, Central Jakarta, Jakarta, Indonesia

First, we thank the reviewer for this insightful comment. We have explained the rationale for choosing Tenganan and Wanagiri in the context section. However, in accordance with the reviewer's questions, we have revised the article. It now reads:

 “The study was conducted in two villages, namely Tenganan Village and Wanagiri Village. The two villages were chosen because: 1) Both villages represent two dominant management models. The forest land in Tenganan is communally owned, which is a model of prototypically traditional customary management, while the forest land in Wanagiri Village is state-owned, which is a model of state-supported community forest management; (2) Managers of customary forests and village forests have been incorporated into forest farmer group established before the COVID-19 pandemic. Simply said, forest farmer groups in Tenganan and Wanagiri have been well-established before the COVID-19 pandemic. Therefore, we could observe the resilience of both institutional structures and the behavior of migrant recipient families (especially those who manage customary forest and village forest land); (3) there have been no extreme changes in land cover before, during, and after the COVID-19 pandemic. This condition has allowed us to ascertain the socio-managerial dynamics among managers and migrant recipients of customary forest and village forest land.”

 Second, a variable, which is equally known as a concept or construct, is a symbol assigned a numerical or other value (Kerlinger, 1973). In the same book, it is stated that a variable is a property that can have different values ​​(in research, variables must have diverse values). Furthermore, complying with this suggestion, we draw an analogy with religiosity. Religiosity is defined as having a strong belief in God or gods. According to the Indonesian Dictionary (KBBI), religiosity is devotion to religion; piety. A person's religiosity is not binary (simply whether someone is "religious" or "not religious"), but rather it is a continuum that extends from low to high degrees. Such a variation has allowed the researchers to analyze its relationship with other variables. In other words, religiosity is a measurable variable in research because it qualifies an attribute with varying values ​​and can be operationalized through measurable dimensions. Religiosity has several measurable variable dimensions: Attachment to God, Trust-Mistrust in God, Daily Spiritual Experience, Religious Coping, Faith Maturity, Religious History (Koenig et al., 1999). Likewise, Tri Hita Karana (THK) is defined as three sources of happiness. In this study, THK refers to the level of respondents' internalization of the Human-God, Humans-Humans, and Humans-Nature dimensions. In accordance with the reviewer's questions, we have revised the article. It now reads:

 “As with the general application of TPB theory, individual serves as the unit of analysis in this study. THK is defined as the level of respondent's internalization of the Human-God, Humans-Humans, and Humans-Nature dimensions, as measured with the respondent's (individual's) subjective perception. Therefore, THK has different values ​​for different individuals. Figure 3(B) illustrates that THK represents a harmonious integration of three related realms: the human world (pawongan), the natural world (palemahan), and the spiritual world (parahyangan). The self (microcosm) is indispensable from the universe (macrocosm) and both are composed of the same elements (Adityanandana and Gerber 2019). Furthermore, Citing Flood (1997) and Agung (2005), they explained that in THK, violence against nature is self-harming. Nature is believed to be a manifestation of the Supreme Being, and accordingly, is fundamentally sacred. Nature is not a merely human-exploited object, but instead, it deserves respect, as captured in the section Svet a svataropanisad: Submit to God because God is in the fire, in the air, in the entire universe, in the plants above the trees”. The THK concept of the spiritual world is relevant to the doctrine of entelechy. This doctrine is classically outlined in Purusartha (the object of human pursuit), which consists of moral (dharma), economic (artha), sensual (kama), and spiritual (moksha) values. In spite of their whole importance, the pursuit of economic and sensual desires should not override moral values ​​in order to achieve spiritual fulfillment (the ultimate goal—moksha). The path to moksha (or liberation from suffering, inherent in the cycle of rebirth) unfolds over several lifetimes depending on one's actions (karma) and the soul's maturity to detach itself from the physical world.”

 Third, we thank the reviewer for this insightful comment. To deal with this issue, we present the history of the TPB. The TPB is an extension of the TRA (Traditional Modeling Model). TRA was developed to better understand the relationship between attitudes, intentions, and behavior (Fishbein, 1967). Some researchers have used TRA to explain and predict individual behavior. However, the results are not always satisfactory because TRA has limited power in predicting behavior beyond the individual's control. Simply stated, TRA assumes that humans have a complete volitional control over their actions. However, in reality, many such external factors as money, time, skills, or legal regulations effect human behaviors. TRA generally fails to predict the action when someone expects to do something yet lack the resources. In attempt to deal with this shortcoming, Ajzen added the variable Perceived Behavioral Control (PBC). This variable accommodates both real and perceived barriers that a person face up. PBC is a person's perception of the level of ease or difficulty to perform a particular behavior. This variable accommodates both real and perceived barriers faced by a person. PBC has two main components, namely: (1) Control Beliefs: Beliefs about the existence of factors that can facilitate or hinder the performance of a behavior (for example: availability of time, money, or skills); and (2) Perceived Power: An individual's assessment of how strongly these supporting or inhibiting factors affect their ability to act. Thus, formal rules, customary law, or economic necessity have been summarized in PBC.

 Nevertheless, from the proposed model (Figure 3C), THK affects behavioral intentions through attitudes, subjective norms, and PBC. Stated in a different way, THK affects attitudes and subjective norms, which has different values ​​across individuals. In accordance with the reviewer's questions, we have revised the article. It now read:

 “This study reveals how the COVID-19 pandemic has not had a significant impact on forest management specially on Tenganan forests and Wanagiri forests in Bali. This study has presented an enhanced version of the TPB model that adds a new variable called THK to explain the behavior of migrant-receiving families in order to provide an answer to the condition. The conclusion is that THK, which is a value in Balinese society, is an antecedent predictor of behavioral intention to manage forests sustainably, mediated by attitude, SN, and PBC.

 The effect of THK on behavioral intentions is mediated by subjective norms. This suggests that THK functions as a collective moral compass rather than merely an individual belief. In the Balinese context, the THK philosophy is embedded in social expectations. Therefore, individuals are socially enforced to practice sustainable forest management by maintaining harmony with their community (Pawongan) and their environment (Palemahan), as expected by their social environment.

 THK significantly affects behavioral intentions through the mediation of perceived behavioral control. This suggests that THK values — particularly social cohesion found in Pawongan (formal rule, customary law, or economic necessity)—strengthen individuals' sense of independence and self-efficacy. THK has improved respondents’ sense of empowerment and perceptually reduced barriers to practice sustainable forest management, as they relied on the collective strength and traditional institutional support inherent in the THK framework.”

 Fourth, we appreciate the reviewer’s emphasis on model stability. Nevertheless, the Inverse Square Root (ISV) and Exponential Gamma (EG) methods are primarily designed for research of large target population. Both ISV and EG are applied to determine the minimum sample size in research, especially in Structural Equation Modeling, where the target population is large. These methods were developed to: (1) overcome the limitations of the “10-fold rule of thumb” which often results in inaccurate sample size estimates in more complex models; and (2) ISR and EG are particularly useful in a large target population research, but require a more efficient and accurate minimum sample size estimate based on the lowest R2 value and the lowest path coefficients in the model (Kock & Hadaya, 2018). Meanwhile, the aim of this study is to gain improved understanding of the behavior of migrant-receiving families. The word "migrants" in this study exclusively refers to return migrants, namely the inevitably laid off workers due to COVID-19. This study focuses on the hometown of the migrants by focusing on the behavior of migrant-receiving families (especially managers of customary forests and village forests). The research objectives and focus above clearly defines that the target population is specific (forest management families who foster the laid-off relatives due to COVID-19).  Therefore, in our opinion, ISR and EG are not relevant. However, in the article, we have searched for and written the GoF value. The benefits of the GoF value are: (1) Assessing the Predictive Ability of the Model; (2) Preventing Information Failure; and (3) Providing Legitimacy to the Model (Parsimony). In accordance with the reviewer's questions, we have revised the article. It now reads:

 “Before testing the hypothesis, we calculate the Goodness-of-Fit (GoF). Overall, GoF should be the starting point for model assessment. If the model does not fit the data, then the data contains more information than the model can convey (Henseler et al. 2016). The benefits of GoF are: (1) Assessing Model Predictive Ability. The main benefit of GoF is that it is a single index to evaluate the overall quality of both measurement models and structural models (Tenenhaus et al., 2005); (2) Preventing Information Failure. GoF serves to ensure that the model does not "throw away" important information contained in the data. Lack of fitness of the model will result in debatable conclusions (Henseler et al. 2016), (3) Providing Legitimacy to the Model (Parsimony). GoF is beneficial to provide evidence the simple model can be very effective in explaining complex phenomena (Wetzels et al. 2009). The GoF value in this study is 0.685. Referring to Henseler et al.'s (2016) criteria, this value exceeds the threshold for the large category, indicating that the proposed model has high consistency with the data. Therefore, the proposed model is consistent with the data, and the tested model is parsimonious and reasonable.”

 Fifth, in accordance with the reviewer's questions, we have revised the article. It now reads:

 “The non-probabilistic sampling strategy is applied in this study to acknowledge several limitations to the external validity of the findings. Rather than offering universal generalizations across all social forestry models, the results of this study apply only to villages with similar socio-ecological profiles. In particular, these findings represent autonomously and traditionally managed by villages based on customary law (awig-awig), with communal land ownership, and the state-owned forests managed collaboratively by the Village Forest Management Institution and the government, namely the Ministry of Environment and Forestry.”

# Reviewer report: Reviewer response for version 3

**DOI:** 10.5256/f1000research.184518.r398893

**Published:** 2025-07-21

**Authors:** James Roshetko

**Affiliations:** 1 The World Agroforestry Centre (ICRAF), Bogor, West Java, Indonesia

It looks good. No further comments.

Is the work clearly and accurately presented and does it cite the current literature?

Partly

If applicable, is the statistical analysis and its interpretation appropriate?

I cannot comment. A qualified statistician is required.

Are all the source data underlying the results available to ensure full reproducibility?

Yes

Is the study design appropriate and is the work technically sound?

Yes

Are the conclusions drawn adequately supported by the results?

Partly

Are sufficient details of methods and analysis provided to allow replication by others?

Yes

Reviewer Expertise:

NA

I confirm that I have read this submission and believe that I have an appropriate level of expertise to confirm that it is of an acceptable scientific standard.

# Reviewer report: Reviewer response for version 2

**DOI:** 10.5256/f1000research.181846.r386565

**Published:** 2025-06-11

**Authors:** James Roshetko

**Affiliations:** 1 The World Agroforestry Centre (ICRAF), Bogor, West Java, Indonesia

The paper is interesting and contributes to the existing literature. It requires revision regarding: consistent use of terminologies; a broad review of relevant literature; and expansion of the Discussion to directly address 'sustainable management of customary and village forests' in the context of return migrants. 

 Specific comments/recommendations:

1. Title: The title should reflect the focus of the study on the effect of ‘return migrant’ on sustainable forest management.
2. Two sets of aims are stated in the paper, one on page 5 and another on page 6. They are not consistent. See possible edit to clarify the situation.
3. As stated, the second aim (page 6) is ‘to improve understanding of the behavior of migrant receivers’. What is a ‘migrant receiver’? That term is never used again.  Is it the same as ‘migrant-receiving family’? Be consistent in terminology.
4. In the paper does the term ‘migrant’ always refer to ‘return migrant’? If yes, include a statement making that point in the Introduction (after the aim). Currently there is a lack of clarity.
5. Page 5. The Introduction could include a broader review of the literature, as the impact of COVID on local NRM varied. An example Utomo et al (2022) provides both support and contradiction to the current Introduction. Utomo et al. (2022) found COVID caused reduced production of bamboo crafts with a corresponding reduction of income in the bamboo craft sector. Response to that situation included an increase in the utilization of local forest resources (increased hunting and gathering) as well as an increase in farming. Citation and link here. The authors may know other relevant literature.
  - Utomo MMB, et al., 2022 (Ref 1)
6. Page 5. Second ‘aim’ – clarify the field data collected.
7. Page 6. The authors should provide definitions of or distinguish the difference between 'customary forests' and 'village forests', in the context of this research.
8. Page 6. It would be good to include the Indonesia terminology of the 3Ms - memakai masker, mencuci tangan, menjaga jarak.
9. Page 7. Be consistent in use of capitalization. Throughout the paper.
10. Page 10. Be consistent in the use of terminology. The Introduction gives the ‘aims’ of the study. Page 10 refers to the ‘objectives’ of the study.
11. Page 10. There should only be one decimal place in the % data. 97.2% and 2.8%. Adopt this standard throughout the paper. There is an exception on Page 6. Round the data appropriately.
12. Page 13. It is ok to provide an example from China (Gou et al 2022). It is much more relevant to cite cases from Indonesia. The authors should include relevant Indonesian cases.
13. Page 13. The authors stated 'many literatures' than cite only two studies by the same authors. That is not 'many'. Suggest the authors add studies that show a time progression from 2000 to 2020. Additionally, in the last sentence state clearly why the 'layoffs and return migration' increased deforestation pressure.
14. Pages 13 and 14.  The Results and Discussion section largely focuses on the function of the TPB framework with linkage to THK principles. Currently there are only vague statements regarding 'continuing sustainable forest management'. Presenting the ‘sustainable forest management’ data/results in Table 1 through 4 is not adequate. In the Discussion there should be a summary of the results regarding respondents' views of 'sustainable management of customary and village forests' in the context of 'return migrant receiving families'. Sustainable customary and village forest management is a dominant term in the title of the article - but it is not addressed in the Discussion
15. Discussion. In the Discussion I anticipate review/discussion of ‘community/social forestry’ issues that are relevant to the ‘returned migrants’ impact on traditional management.  The authors may consider adding such analysis, which would make the paper pertinent to a wider range of cases and research. The authors doubtlessly are familiar with relevant literature. Some general options from the global, regional and country level are:
  -Gilmour DA. 2016. Forty years of community based and effectiveness. Rome: Food and Agriculture Organization (FAO). http://www.fao.org/3/a-i5415e.pd- Wong GY, et al., 2020 (ref 2)
  -De Royer S, et al., 2018 (Ref 3)
16. Minor edits are suggested throughout the paper to improve grammar style, clarity and reduction of superfluous text.

Is the work clearly and accurately presented and does it cite the current literature?

Partly

If applicable, is the statistical analysis and its interpretation appropriate?

I cannot comment. A qualified statistician is required.

Are all the source data underlying the results available to ensure full reproducibility?

Yes

Is the study design appropriate and is the work technically sound?

Yes

Are the conclusions drawn adequately supported by the results?

Partly

Are sufficient details of methods and analysis provided to allow replication by others?

Yes

Reviewer Expertise:

smallholder agroforestry systems, smallholder timber production, smallholder commodity production, germplasm quality, smallholder nurseries, livelihood enhancement, and climate smart agroforestry

I confirm that I have read this submission and believe that I have an appropriate level of expertise to confirm that it is of an acceptable scientific standard, however I have significant reservations, as outlined above.

## References

[1] : COVID-19 Pandemic: Impacts, Craftsmen’s Living Strategies, and Economic Recovery of Bamboo Handicraft Enterprise in Gunungkidul, Indonesia. *Forest and Society* .2022;6(2) : 10.24259/fs.v6i2.20599 10.24259/fs.v6i2.20599

[2] : Social forestry in Southeast Asia: Evolving interests, discourses and the many notions of equity. *Geoforum* .2020;117: 10.1016/j.geoforum.2020.10.010 246-258 10.1016/j.geoforum.2020.10.010

[3] : Does community-based forest management in Indonesia devolve social justice or social costs?. *International Forestry Review* .2018;20(2) : 10.1505/146554818823767609 167-180 10.1505/146554818823767609

# Response

**Published:** 2025-07-04

**Authors:** Kresno Agus Hendarto

**Affiliations:** Pusat Riset Kependudukan, Badan Riset dan Inovasi Nasional Republik Indonesia, Central Jakarta, Jakarta, Indonesia

1. Title: The title should reflect the focus of the study on the effect of ‘return migrant’ on sustainable forest management.

*Thank you for suggestion. It currently reads as follows:*

*An extended Theory of Planned Behavior in explaining intention toward sustainable forest management: Evidence from COVID 19 Pandemic from Bali, Indonesia.*

 2. Two sets of aims are stated in the paper, one on page 5 and another on page 6. They are not consistent. See possible edit to clarify the situation.

*Thank you for suggestion. It currently reads as follows:*

*Reviewing these literatures, we found that significant gap, especially on migrant-receiving family. (1) most of the studies were based on assumptions (useful for scenario analysis) and generally, they used secondary data or online survey, instead of the actual data taken in the field; (2) …*

*Thus, the aim of this study is to improve understanding of the behavior of the migrant-receiving families.*

 3. As stated, the second aim (page 6) is ‘to improve understanding of the behavior of migrant receivers’. What is a ‘migrant receiver’? That term is never used again.  Is it the same as ‘migrant-receiving family’? Be consistent in terminology

*Thank you for suggestion. It currently reads as follows:*

*Migrant receiver here is the same as migrant-receiving family.*

 4. IIn the paper does the term ‘migrant’ always refer to ‘return migrant’? If yes, include a statement making that point in the Introduction (after the aim). Currently there is a lack of clarity.

*Thank you for suggestion.*

*Migrants in this study refer to return migrants.*

*It currently reads as follows:*

*Thus, the aim of this study is to improve understanding of the behavior of the migrant-receiving families. The word migrants in this study exclusively refers to return-migrant, namely those who inevitable return to their hometowns as a result of layoffs due to COVID-19. In other words, this study aims to investigate of the insignificant impact of COVID-19 pandemic-related damage on the management of customary forests and village forests in Bali.troduction (after the aim). Currently there is a lack of clarity.*

 5. Page 5. The Introduction could include a broader review of the literature, as the impact of COVID on local NRM varied. An example Utomo et al (2022) provides both support and contradiction to the current Introduction. Utomo et al. (2022) found COVID caused reduced production of bamboo crafts with a corresponding reduction of income in the bamboo craft sector. Response to that situation included an increase in the utilization of local forest resources (increased hunting and gathering) as well as an increase in farming. Citation and link here. The authors may know other relevant literature.

 - Utomo MMB, et al., 2022 (Ref 1)

*Thank you for suggestion. It currently reads as follows:*

*During COVID-19, bamboo craftsmen in Gunung Kidul Yogyakarta have earned up to 23.5% lower income. Consequently, they do such other jobs as farming, hunting, and selling food (Utomo et al. 2022). Likewise, a systematic evaluation using 62 indicators has revealed that COVID 19 has caused a significant decrease in production marketing, income, and social interaction for forest farmers in Sikka, East Nusa Tenggara (Njurumana et al. 2025).*

 6. Page 5. Second ‘aim’ – clarify the field data collected.

*Thank you for suggestion. It currently reads as follows:*

*Second, this study gathers field data. This study collects information directly from the source to ensure more authentic and representative insights, which can objectively reveal real-world phenomena and capture the possibly overlooked complexities that are relevant to the study questions or hypotheses.*

 7. Page 6. The authors should provide definitions of or distinguish the difference between 'customary forests' and 'village forests', in the context of this research.

*Thank you for suggestion. It currently reads as follows:*

*The Regulation of the MoEF Number 9 of 2021 concerning social forestry management stated that customary forests are located within the territory of customary law communities. Customary areas refer to lands and/or waters along with the natural resources thereon, that have certain boundaries, and are owned, utilized preserved, and sustained from generation to generation to meet the needs of the community. The customary lands or forests are inherited from ancestors or acquired through the claim of ownership…*

*The Village forest is a forest areas that have not been burdened with permits. The village forests are customarily managed and utilized by the village for the welfare of the village, village areas, or areas resulting from management boundary agreements between adjacent villages. The community maps them in a participatory manner, and/or located within a single natural landscape in the village.*

 8. Page 6. It would be good to include the Indonesia terminology of the 3Ms - memakai masker, mencuci tangan, menjaga jarak

*Thank you for suggestion. It currently reads as follows:*

*In order to protect the public’s health during the COVID 19 epidemic, the government imposed travel restrictions, promoted the 3M campaigns (Memakai masker, Mencuci tangan, dan Menjaga jarak/ mask use, hand washing, and maintaining social distancing), and administered vaccinations.*

 9. Page 7. Be consistent in use of capitalization. Throughout the paper

*Thank you for suggestion.*

*We have carefully corrected the use of capitalization throughout the paper.*

 10. Page 10. Be consistent in the use of terminology. The Introduction gives the ‘aims’ of the study. Page 10 refers to the ‘objectives’ of the study.

*Thank you for suggestion. It currently reads as follows:*

*Following of the study aims, the specific criteria are people who: (1) cultivators of customary forests and village forests; (2) …*

 11. Page 10. There should only be one decimal place in the % data. 97.2% and 2.8%. Adopt this standard throughout the paper. There is an exception on Page 6. Round the data appropriately

*Thank you for suggestion. It currently reads as follows:*

*Comparing the same period in 2019 to all tropical areas, deforestation increased by 63.0% to 136.0% ( Brancalion et al. 2020).*

*The open unemployment rate in February 2020 was 1.3%, which increased to 5.4% in February 2021 and fell slightly to 5.3% in February 2022. The poverty rate increased, in March 2020 it was 165.19 (3. 8%) while in March 2021, it was 201.97 (4.5%) ( BPS 2022).*

*During COVID-19, bamboo craftsmen in Gunung Kidul Yogyakarta have earned up to 23.5% lower income. Consequently, they do such other jobs as farming, hunting, and selling food (Utomo et al. 2022).*

*For example, forest cover on Mansinam Island decreased by 4.3%, wasteland increased by 80.6%, agricultural land increased by 75.3%, and shrubs increased by 54.9%. Another finding is that 78.9% of total deforestation has resulted from forest conversion to wasteland and agricultural land ( Hematang et al., 2025).*

*The total population in the village of Wanagiri is 4,056 people; 51. 6% of which are men and 48.4% are women. Religion of the population are Hindus (98.9%); Islam (0. 7%); Christian (0.2%); Catholic (0.1%); and Budhis (0.1%) ( Sistem_Informasi_Desa, 2023).*

*Of the 71 data analyzed, the respondents were born and raised in the villages of Wanagiri and Tengganan (97.2%) while the rest were not born in the location and live in the villages of Wanagiri and Tengganan due to marriage (2.8%).*

*Most of the village forests have been planted with coffee. Total population in the village of Wanagiri is 4,056 people; 51.6% of which are men and 48.4% are women. Religion of the population are Hindus (98.9%); Islam (0.7%); Christian (0.2%); Catholic (0.1%); and Budhis (0.1%) (Sistem_Informasi_Desa, 2023). There are 296 families involved in village forest management. This number is divided into 3 forest farmer groups, namely Wana Amerta (with 78 families); Puncak Manik (35 families); and Jagra Wana (78 families).*

*Therefore 71 questionnaires were analyzed. Of the 71 data analyzed, the respondents were born and raised in the villages of Wanagiri and Tengganan (97.2%) while the rest were not born in the location and live in the villages of Wanagiri and Tengganan due to marriage (2.8%). Table 1 contains information about the respondent’s profile.*

*It implies an income discrepancy of IDR 147,344,000 or a decrease of up to 30.0% (Widhanarto et al. 2024).*

 12. Page 13. It is ok to provide an example from China (Gou et al 2022). It is much more relevant to cite cases from Indonesia. The authors should include relevant Indonesian cases.

*Thank you for suggestion. It currently reads as follows:*

*For the case in Indonesia, there was a decrease in income in the wood processing industry business in Labe Lawe, Sekadau Hilir Regency, West Kalimantan, from previously IDR 492,927,000 before the Covid-19 pandemic and IDR 345,583,000 during the Covid-19 pandemic. It implies an income discrepancy of IDR 147,344,000 or a decrease of up to 30.0% (Widhanarto et al. 2024).*

 13. Page 13. The authors stated 'many literatures' than cite only two studies by the same authors. That is not 'many'. Suggest the authors add studies that show a time progression from 2000 to 2020. Additionally, in the last sentence state clearly why the 'layoffs and return migration' increased deforestation pressure

*Thank you for suggestion. It currently reads as follows:*

*Literatures reveales that deforestation (pressure on forests) has resulted from the increased conversion of forest land when compared to the desire to control the trees ( Kaimowitz & Angelsen 1998; Angelsen & Kaimowitz 1999). In general, this conversion aims at opening up land for industry, settlements, plantations, agriculture, mining and others. When the COVID-19 pandemic occurred, this pressure increased with layoffs and return migration. When layoffs hit, they generally return to their hometowns to seek new opportunities, find temporary housing, and/or look for emotional and social support.. On the other hand, their hometowns offer very few formal employment opportunities that match the skills of the return migrants. Reduced or without income will force the return-migrant to turn to natural (agricultural) resources for survival. This will subsequently put pressure on the existing agricultural land, which in turn will encourage return-migrant to engage in activities that exploit natural resources including forests.*

 14. Pages 13 and 14.  The Results and Discussion section largely focuses on the function of the TPB framework with linkage to THK principles. Currently there are only vague statements regarding 'continuing sustainable forest management'. Presenting the ‘sustainable forest management’ data/results in Table 1 through 4 is not adequate. In the Discussion there should be a summary of the results regarding respondents' views of 'sustainable management of customary and village forests' in the context of 'return migrant receiving families'. Sustainable customary and village forest management is a dominant term in the title of the article - but it is not addressed in the Discussion

*Thank you for suggestion.*

*In line with the reviewer's suggestion and our response to point 1, we can explain this as follows:*

*The main objective of the study entitled Extended Theory of Planned Behavior (TPB) is to add new variables (constructs) to the original TPB model to improve the predictive and explanatory ability of human behavior. The original TPB model is available in Figure 3a. Although TPB has been proven effective in predicting various behaviors, researchers have recognized other influencing factors of intentions and behaviors as having not been included in the original model. In other words, the purpose this study is to build an accurate model in explaining and predicting the behavior of migrant-receiving families by including additional relevant contextual factors (i.e. Tri Hita Karana as a value).*

 15. Discussion. In the Discussion I anticipate review/discussion of ‘community/social forestry’ issues that are relevant to the ‘returned migrants’ impact on traditional management.  The authors may consider adding such analysis, which would make the paper pertinent to a wider range of cases and research. The authors doubtlessly are familiar with relevant literature. Some general options from the global, regional and country level are:

 -Gilmour DA. 2016. Forty years of community based and effectiveness. Rome: Food and Agriculture Organization (FAO). http://www.fao.org/3/a-i5415e.pd- Wong GY, et al., 2020 (ref 2)

 -De Royer S, et al., 2018 (Ref 3)

*Thank you for suggestion. It currently reads as follows:*

*Social forestry often fails to provide full rights to local communities, especially in the context of customary rights and formal recognition (Wong et al. 2020). This is particularly true because participation is limited to village elites and technical support is almost non-existent. In other words, the main obstacles include bureaucratic processes and local actor exclusion (Royer et al. 2018). However, Decree number 1546 / MenLHK-PSKL / PKTHA / Kum.1 / 2/2019 of the Minister of Environment and Forestry (KLHK) that determined the forest in Tenganan as a customary forest; Decree of the Governor of Bali Number 2017 / 03-L / HK / 2005 that determined it as a Village Forest in Wanagiri; and Decree of the Governor of Bali Number 2017 / 03-L / HK / 2015 as well as Decree of the Buleleng Regent Number 430/405 / HK / 2017 concerning the Management of Tourism Villages in Wanagiri, have reduced some of these detrimental factors. This aligns with social forestry’s key success factors (Gilmour 2016): secure tenure rights, supportive regulation, strong governance, market access, and bureaucratic support.*

*The residents of Wanagiri Village have established a tourism awareness group as a part of the village-owned enterprise (BUMDes). Their income is derived from managing local natural tourist attractions such as Banyumala Waterfall, Puncak Manik Waterfall, and Buana Sari Waterfall, as well as providing tour guides (Laksemi et al., 2019). Local entrepreneurship practices in Wanagiri Village are organized by the BUMDes “Eka Giri Karya Utama”. The economic programs include savings and loans, tourism, coffee processing, waste management, and water management. They also developed LPD (Village Credit Institution) a village-level credit institution to provide financial access.*

*Likewise, the residents of Tenganan Village maintain the customary system and local wisdom in forest management. The community applies awig-awig to maintain and sustain the forest, even before receiving formal recognition from the government. These awig-awig are customary regulations that contain recommendations and prohibitions (for example, the prohibition of cutting down trees and changing the function of forest land without permission from the Traditional Village). The highly obedient community reflects the effectiveness of the awig-awig. Most resident whose lives are highly dependent on the sale of commodities, especially palm leaf crafts, Gringsing cloth (whose materials are collected from customary forests) stated that the tourism industry in Tenganan Village is very important and needs to be continuously developed. However, some indigenous people perceive tourism development as a mere bonus of the strengthening of local culture.*

*The two cases showed that SF in Wanagiri is more proactive in articulating ideas and social participation in village deliberation forums, while SF in Tenganan has provided full rights to the local community.*

 16. Minor edits are suggested throughout the paper to improve grammar style, clarity and reduction of superfluous text.

*Thank you for suggestion.*

*We have done both using proof reading from Yogyakarta State University and Grammarly.*

# Reviewer report: Reviewer response for version 1

**DOI:** 10.5256/f1000research.174043.r346466

**Published:** 2025-01-30

**Authors:** Sudirman Daeng Massiri

**Affiliations:** 1 Tadulako University, Palu City, Indonesia

The Covid 19 Pandemic problem has passed 5 years ago and Indonesia has successfully overcome this problem. However, this research predicts the impact of forest sustainability caused by the Covid 19 pandemic, through the application of the Theory of Planned Behaviour (TPB) framework. The application of the TPB theoretical framework in predicting the behavior and intention to manage forests is the novelty of this research. 

**Background**

 Authors also need to corroborate with previous research to strengthen whether there is an impact of covid 19 on forest sustainability or on land cover change.

**Sample**

 Why were non-migrant recipient communities not interviewed as behavioral controls?. Is there a difference between the community receiving covid 19 migrants and non-migrant recipients?.

**Discussions**

 Discussions Should  strengthening references that confirm whether the covid pandemic increased pressure on forest destruction? Or whether during covid 19 there was an increase in forest product commodities? 

 The author should also clarify whether there is a difference in the behavior of communities that accept migrants and communities that do not accept migrants.

Is the work clearly and accurately presented and does it cite the current literature?

Yes

If applicable, is the statistical analysis and its interpretation appropriate?

Yes

Are all the source data underlying the results available to ensure full reproducibility?

Yes

Is the study design appropriate and is the work technically sound?

Yes

Are the conclusions drawn adequately supported by the results?

Yes

Are sufficient details of methods and analysis provided to allow replication by others?

Yes

Reviewer Expertise:

Forest Policy, Social forestry

I confirm that I have read this submission and believe that I have an appropriate level of expertise to confirm that it is of an acceptable scientific standard, however I have significant reservations, as outlined above.
