# Supplementary material for: An extended Theory of Planned Behavior in explaining intention toward sustainable forest management: Evidence from COVID 19 Pandemic from Bali, Indonesia
Source: F1000Res. 2025 Jul 17;13:1457. Originally published 2024 Dec 2. [Version 3] doi: 10.12688/f1000research.158455.3 (PMC12831049; doi:10.12688/f1000research.158455.3)
Supplement: Peer review history

# Response

**Published:** 2025-05-08

**Authors:** Kresno Agus Hendarto

**Affiliations:** Pusat Riset Kependudukan, Badan Riset dan Inovasi Nasional Republik Indonesia, Central Jakarta, Jakarta, Indonesia

We appreciate your careful examination of our paper (Submission ID: 158455). We already have made the following changes

 . 

 First, for background section; We have made revisions and added noted. It currently reads as follows:

 Using web-based bibliometric analysis (biblioshiny) on the impact of the COVID-19 pandemic on the forestry sector, Jayasundara et al., (2024) identified that “deforestation” appears most frequently. Subsequently, "conservation", "management", "urban", "recreation", "wildlife", "market", "product", "climate change", "ecosystem services" come next. It is interesting that there has been an increase in forest recreation (especially urban forests) in such developed countries as America (Grima et al., 2020; Ferguson et al., 2022), Japan (Yamazaki et al., 2021), Germany (da Schio et al., 2021). Urban forests have been favored because of they are close to their homes. In addition to urban forests, undeveloped forests (wilderness areas) are other favorite destinations since the exercise of social distancing regulations. Therefore, they visit isolated tourist locations with only few visitors. On the other hand, the number of retiree visitors (who are older), international visitors, and those from distant locations has decreased. Likewise, the intensity of activities forest conservation and management has decreased because in general the government has allocated the budget mostly to deal with COVID-19. In other words, the budget for conservation and forest management activities has been cut significantly. Consequently, such illegal activities as deforestation, land encroachment, and poaching have increased (Brancalion et al., 2020). With the transit point in Bangladesh, Rahman et al., (2021) revealed that, hunting and trade of wild animals has increased in India, Nepal, Myanmar, Thailand and China. The people whose lives depend on forests face serious challenges. The restrictions imposed has resulted in income decline. For instance, in Indonesia, limited accessibility has resulted in the decline in honey harvest and income(Njurumana et al., 2021). The decline in demand for agroforestry products has consequently resulted in reduced income (Pieter et al., 2022). The decline of ecotourism due to the decrased number of distant and international visitors has decreased the income of the communities around ecotourism locations (Kalema-zikusoka, 2021; Rahaman et al., 2021). Furthermore, research on forest cover showed that during the pandemic tropical forest cover on small inhabited islands has declined. For example, forest cover on Mansinam Island decreased by 4.3%, wasteland increased by 80.6%, agricultural land increased by 75.3%, and shrubs increased by 54.9%. Another finding is that 78.9% of total deforestation has resulted from forest conversion to wasteland and agricultural land (Hematang et al., 2025)

 Second, for sampling methodology: We use surveys to collect data. Surveys (non-experiments) are different from experiments. In experimental research we classify a small number of people into equal treatment and control groups, test one or two hypotheses, and manipulate conditions to minimize internal validity. Quantitative data are obtained at the end of the experiment and participants' responses are compared to the dependent variable. On the other hand, survey research has a different mechanism.  Usually a large sample of respondents are contacted and asked about the same questions. Multiple variables are measured and hypotheses are tested simultaneously. Tentative inferences are concluded from questions about past behavior, experiences or characteristics. Surveys are sometimes called correlational because researchers do not control and manipulate conditions as in experiments (Neuman, 2019). The most distinguishing characteristic of experimental research from survey research is the way in which the independent variable is created rather than simply measured (Zikmund & Babin, 2016).

 Ex post facto research is different from experiments. Experiments involve the manipulation of independent variables by the researcher, while ex post facto methods involve researchers examining past events to determine their causes. Some strategies have been adopted for increasing the credibility of ex post facto research, although none of them can adequately overcome the basic weakness of this research, namely the lack of control over the independent variables. These strategies include matching, analysis of covariance and partial correlation, homogeneous groups, and building extraneous variables into the design (Sappaile, 2010).

 This study is controlled by using a "homogeneous group". Collecting heterogeneous samples and comparing the subgroups in them (which must be matched) will make it difficult to determine the time frame. The longer the time frame, the farther it will be from the objective of this article. Therefore, samples were exclusively collected from homogeneous subjects (who received migrants due to the COVID-19 pandemic)

 Third, for discussion section, We have made revisions and added noted. It currently reads as follows:

 When the COVID-19 pandemic first occurred in Italy, demand for wood contracted. The international market (export-import) of wood products experienced a sharp decline (Barcaccia et al., 2020). Likewise in China, the price of natural resource commodities is volatile and short termed due to the disrupted supply and demand chains resulting from the increase in active cases and the spike in deaths of COVID-19 patients (Guo et al., 2022). By comparing the impact of COVID-19 to that of previous economic crises, (Wunder et al., 2021) found that national income and commodity prices are affected by 3 factors, namely: contractionary-inflationary supply-side shocks, deflationary demand-side impacts, and expansionary-inflationary government policy responses (both monetary and fiscal). In the US, the COVID-19 pandemic caused a significant shift in the demand function, while the supply function shifted inward due to labor shortages. The lumber price in the US increased from $319.70 per thousand board feet (mbf) in April 2020 due to the appearing sign of pandemic resolution. It reached the peak of $1500.50/mbf exactly one year later(van Kooten & Schmitz, 2022).

 Many literatures revealed that deforestation (pressure on forests) has resulted from the increased conversion of forest land when compared to the desire to control the trees (Kaimowitz & Angelsen, 1998; Angelsen & Kaimowitz, 1999).  In general, this conversion aims at opening up land for industry, settlements, plantations, agriculture, mining and others. When the COVID-19 pandemic occurred, this pressure increased with layoffs and return migration.
